# Full-sized realistic 3D printed models of liver and tumour anatomy: a useful tool for the clinical medicine education of beginning trainees

**DOI:** 10.1186/s12909-023-04535-3

**Published:** 2023-08-15

**Authors:** Guoqiang Bao, Ping Yang, Jiangpu Yi, Shujia Peng, Jiahe Liang, Yajie Li, Dian Guo, Haoran Li, Kejun Ma, Zhenyu Yang

**Affiliations:** 1 Department of General Surgery, The Second Affiliated Hospital of Air Force Medical University, Xi’an, Shaanxi 710038 China; 2 3D Printing Research Center of Tangdu Hospital, Air Force Medical University, Xi’an, Shaanxi China; 3 Xi ’an Ma Ke Medical Technology Ltd, Room 21516, Block C, Chaoyang International Plaza, Xi’an, Shaanxi China

**Keywords:** 3D-printed models, Simulation-based medical education, Continuing medical education, Liver surgery

## Abstract

**Background:**

Simulation-based medical education (SBME) and three-dimensional printed (3DP) models are increasingly used in continuing medical education and clinical training. However, our understanding of their role and value in improving trainees’ understanding of the anatomical and surgical procedures associated with liver surgery remains limited. Furthermore, gender bias is also a potential factor in the evaluation of medical education. Therefore, the aim of this study was to evaluate the educational benefits trainees receive from the use of novel 3DP liver models while considering trainees’ experience and gender.

**Methods:**

Full-sized 3DP liver models were developed and printed using transparent material based on anonymous CT scans. We used printed 3D models and conventional 2D CT scans of the liver to investigate thirty trainees with various levels of experience and different genders in the context of both small group teaching and formative assessment. We adopted a mixed methods approach involving both questionnaires and focus groups to collect the views of different trainees and monitors to assess trainees’ educational benefits and perceptions after progressing through different training programs. We used Objective Structured Clinical Examination (OSCE) and Likert scales to support thematic analysis of the responses to the questionnaires by trainees and monitors, respectively. Descriptive analyses were conducted using SPSS statistical software version 21.0.

**Results:**

Overall, a 3DP model of the liver is of great significance for improving trainees’ understanding of surgical procedures and cooperation during operation. After viewing the personalized full-sized 3DP liver model, all trainees at the various levels exhibited significant improvements in their understanding of the key points of surgery (p < 0.05), especially regarding the planned surgical procedure and key details of the surgical procedures. More importantly, the trainees exhibited higher levels of satisfaction and self-confidence during the operation regardless of gender. However, with regard to gender, the results showed that the improvement of male trainees after training with the 3DP liver model was more significant than that of female trainees in understanding and cooperation during the surgical procedure, while no such trend was found with regard to their understanding of the base knowledge.

**Conclusion:**

Trainees and monitors agreed that the use of 3DP liver models was acceptable. The improvement of the learning effect for practical skills and theoretical understanding after training with the 3DP liver models was significant. This study also indicated that training with personalized 3DP liver models can improve all trainees’ presurgical understanding of liver tumours and surgery and males show more advantage in understanding and cooperation during the surgical procedure as compared to females. Full-sized realistic 3DP models of the liver are an effective auxiliary teaching tool for SBME teaching in Chinese continuing medical education.

**Supplementary Information:**

The online version contains supplementary material available at 10.1186/s12909-023-04535-3.

## Introduction

In clinical medicine education, learning practical skills is an essential task during medical studies. The use of surgical simulation to reach specific target criteria significantly reduces operation times and improves the performance of surgeons [1]. Simulation-based medical education (SBME) is believed to be superior to the traditional style of medical education according to active and adult learning theories [2]. Furthermore, SBME has the potential to influence students’ motivation and has already been implemented in many medical curricula [3]. In the daily practice of liver surgeons, a wide variety of theoretical, practical and psychosocial competencies must be mastered at a very high level, which can help a surgeon have the ability to perform difficult procedures as early as possible. Thus, the training of these competencies is a central component in the education of liver surgeons to impart what is required for their later practice [4].

With respect to SBME, virtual and augmented reality tools have the potential to offer a more comprehensive alternative to three-dimensional (3D) visualisation but lack the ability to provide tactile feedback [5, 6]. However, 3D printing has proven to be a revolutionary technology in medical education [7–9]. 3D printing, as an additive manufacturing technology based on rapid prototyping, facilitates the creation of patient-specific physical models with high precision [10]; thus, it has been deployed in various medical fields, such as medical education and preoperative planning [11–13]. Additionally, the growing need for tactile and haptic learning in medical education is leading to the increased use of 3D printing in SBME [14, 15]. 3DP models can have both realistic anatomy and textures derived from patients’ individual CT and/or MRI images, thus facilitating the creation of patient-specific physical models with high precision; these models seem to be capable of satisfying the needs for tactile and spatial perception of human anatomical structures [16, 17]. The models can help trainees understand organ physiology, anatomy, tumour characteristics, and surgical procedures more accurately [18]. The application of 3DP models bridges the gap between two-dimensional (2D) imaging and realistic anatomy, as it accurately reproduces anatomical structures and pathologies, thereby providing more tangible information than conventional imaging data [19]. 3DP models also appear to be a significantly more useful and cost-effective technique than traditional cadaveric models in medical education [20], such as those used in undergraduate dentistry training [4], urology residents [21], first-year medical students [22], craniofacial traumas [23], oral and cranio-maxillofacial surgery [24]. However, recent SBME changes regarding 3DP models have focused only on undergraduate medical education [25]. Continuing medical education after postgraduate education has been neglected and under evaluated.

As an intricately vascular architecture and stereoscopic space distribution, the liver is an intriguing yet complex multifunctional organ [26].Despite technological improvements, hepatectomy remains a challenging operation associated with high complication and mortality, especially for young surgeons [27]. Couinaud’s liver segment classification is currently used in liver surgery, but there are many anatomical variations and variables on tumour growth, prior operations, and regenerative growth [28]. Unlike conventional imaging data using 2D films, which can lead to confusion in the actual situation, 3DP liver models are capable of illustrating the relationship between the vascular and biliary channels [29]. 3D anatomical relationships of the vascular and biliary channels are crucial for performing accurate liver resections and predicting the actual size of hepatectomies [30, 31]. This approach may improve residents’ and students’ understanding of organ physiology, anatomy, tumour characteristics, and surgical procedures. Thus, it has the potential to gain increasing importance in the training of residents and students [32, 33], as well as in surgical decision-making and planning for complicated conditions and procedures [34].

According to some studies, gender bias poses a potential threat to the integrity of resident assessment in medical education [35, 36], and gender-related differences are more pronounced among medical students [37, 38], such as visual-spatial working memory [39], spatial learning ability in a virtual environment [40] and confidence [41]. To our knowledge, no study has investigated the educational benefit of 3DP liver models with regard to trainees at different levels in their continuing medical education and trainees of different genders.

### Study goals

Constructivism theory and quantitative post-positivist methods provided a solid theoretical foundation for simulation education in our study. Constructivism theory was proposed by Swiss psychologist Piaget and its core is student-centered, emphasizing students’ active exploration, discovery and construction of the meaning of what they have learned [42, 43]. As the learning environment required by constructivism was strongly supported by the latest information technology achievements, the theory of constructivism has been increasingly combined with the medical education and clinical practice, thus becoming a guiding philosophy for deepening teaching reform in schools [44, 45]. Therefore, we hypothesized that 3DP liver models play a more important role in simulation-based continuing medical education after postgraduate education and may differ according to gender. Accordingly, the aim of the present study was to explore the depth and quality of the 3DP liver models used in the continuing medical education of these different trainees while considering the role of gender by applying SBME educational theory. Understanding the educational benefit of 3DP liver models in continuing medical education can generate evidence that can promote the reform of continuing medical education management programs. Specifically, the objectives of our research on the use of full-sized 3DP live models during trainee education training were as follows:

1) to identify and assess different levels of trainees’ understanding of the relevant base knowledge between the different training models, which is a tool to help trainees better grasp abstract knowledge through spatial visualization.

2) to identify and assess different levels of trainees’ understanding of the key points of surgery between the different training models.

3) to identify the helpfulness and satisfaction of various levels trainees, which were divided into different levels according to when they graduated, including interns (beginner), standardized training trainees (not experienced) and professional training trainees (experienced).

4) to determine whether gender-related differences exist among trainees of continuing medical education, which refers to a specific form of continuing education that helps those in the medical field maintain competence and learn about new and developing areas of their field.

5) to evaluate monitors’ level of satisfaction with the trainees’ cooperation during the operation.

## Materials and methods

### Population and sampling

This was a cross-sectional, analytical and questionnaire-based study. As a preliminary exploratory study, we only surveyed all new trainees at a single centre in the current year, who were enrolled in continuing medical education after their undergraduate course at a large, academic, tertiary care hospital and a university-affiliated medical centre. After we estimated the eligible population in the 10 medical and surgical wards of three teaching tertiary referral hospitals located in the western regions of China, we assumed that 80% of the graduate students will participate and recruited in this study. Based on the predicted anticipated trainees, a minimum sample size of 30 was calculated using the online statistical program Open-epi, taking into consideration a 90% confidence interval, 95% response distribution, 5% margin of error, and an estimated total population size of 70. All participants had completed their internal medicine clerkship rotation at the time of the survey. All participants were at various levels of their training, including interns (beginner), standardized training trainees (not experienced) and professional training trainees (experienced). In the first step, the trainees were divided into 3 groups according to their level of training and sorted by gender (Table 1). Second, all trainees were randomly assigned to one of two groups: one group that received 3D model training and one group that did not.

### Educational context

Based on the syllabus, especially the relevant courses, the basics of liver imaging examinations as well as the physiology and anatomy of the healthy liver were reviewed jointly before continuing the hands-on training. All trainees were given multislice CT/MRI scans and received similar preoperative information on the patients’ disease, tumour characteristics, planned surgery and related risk of complications. This information was delivered during a face-to-face consultation with the supervisors using CT/MRI scan images. Subsequently, the 3D printed models were presented randomly to the assigned trainers in each group. Finally, in this study, half of each group of trainers received a 3D model, while the other half received only 2D images (Table 2). The training occurred 3 times a week, and each session lasted 40 min. After 4 weeks of continuous training, the trainees and the supervisors were required to perform the tasks listed in the questionnaires.

**Table 1:** Classification of the 30 trainees

Group	Gender (Female/Male)			Level of Training	Qualification
A (n = 8)		2/6	Beginner (interns)		No operation attended
B (n = 10)		4/6	Not experienced (standardized training trainees)		No livers operated
C (n = 12)		2/10	Experienced (professional training trainees)		Liver operations assisted

**Table 2:** Classification of Presentation Models

Presentation Models	Description
2D	Conventional 2-D CT scans with or without MRI
3D	Combination of the 3D printed liver models with conventional 2-D CT with or without MRI scan

Two questionnaires were created to prospectively evaluate the trainees’ level of preoperative knowledge and understanding. Their knowledge was first evaluated based on the previously delivered information and CT scan images, following which the 3D printed model was used to assess the improvement following the model presentation. Questionnaire #1 consisted of questions intended to evaluate two components of trainees’ knowledge: (a) basic liver physiology and anatomy, (b) tumour characteristics and (c) questions involved in the planned surgical procedure (“Appendix 1, Table S1”). Questionnaire #2 investigated trainees’ satisfaction using a 10-point Likert scale (“Appendix 2”). Questionnaire #3 was designed to enable supervisors to evaluate the performance of trainees (“Appendix 3”). The other questionnaires were used to evaluate the feedback provided by the trainees with regard to the training models using questions scored on a 10-point Likert scale (“Questionnaire 4, Appendix 4, Table S2”).

### 3D printed model fabrication

#### Imaging data

Considering the cost of the printed model, the imaging data were collected from one patient who had been diagnosed with HCC as well as one healthy volunteer. The imaging data were scanned using a helical Philips iCT256 (Philips Electronics Co., Netherlands). The scanning parameters were 0.6-mm layer thickness, 0.6-mm layer spacing, 120 kV, 280 mAs, and 220 mm field of view (FOV). The contrast agent was iohexol injection 350 (Shanghai GE Pharmaceutical Co., LTD.) at a flow rate of 4.0 ml/s. The original image data were output in the standard digital imaging and communications in medicine (DICOM) format.

### Mimics modeling

The DICOM data were analysed using Mimics to determine whether the scanning range was the anatomical site required by clinical practice. The segment parenchyma, including the artery, liver vein, portal vein, liver, gallbladder, and bile duct, were calculated and exported as STL files using Mimics. Then, procedures such as smoothing, deleting and clipping the model, hollowing out with a certain wall thickness, and designing the connection structure were performed using Magics. A biomedical engineer working alongside surgeons completed model segmentation.

**Fig. 1:**
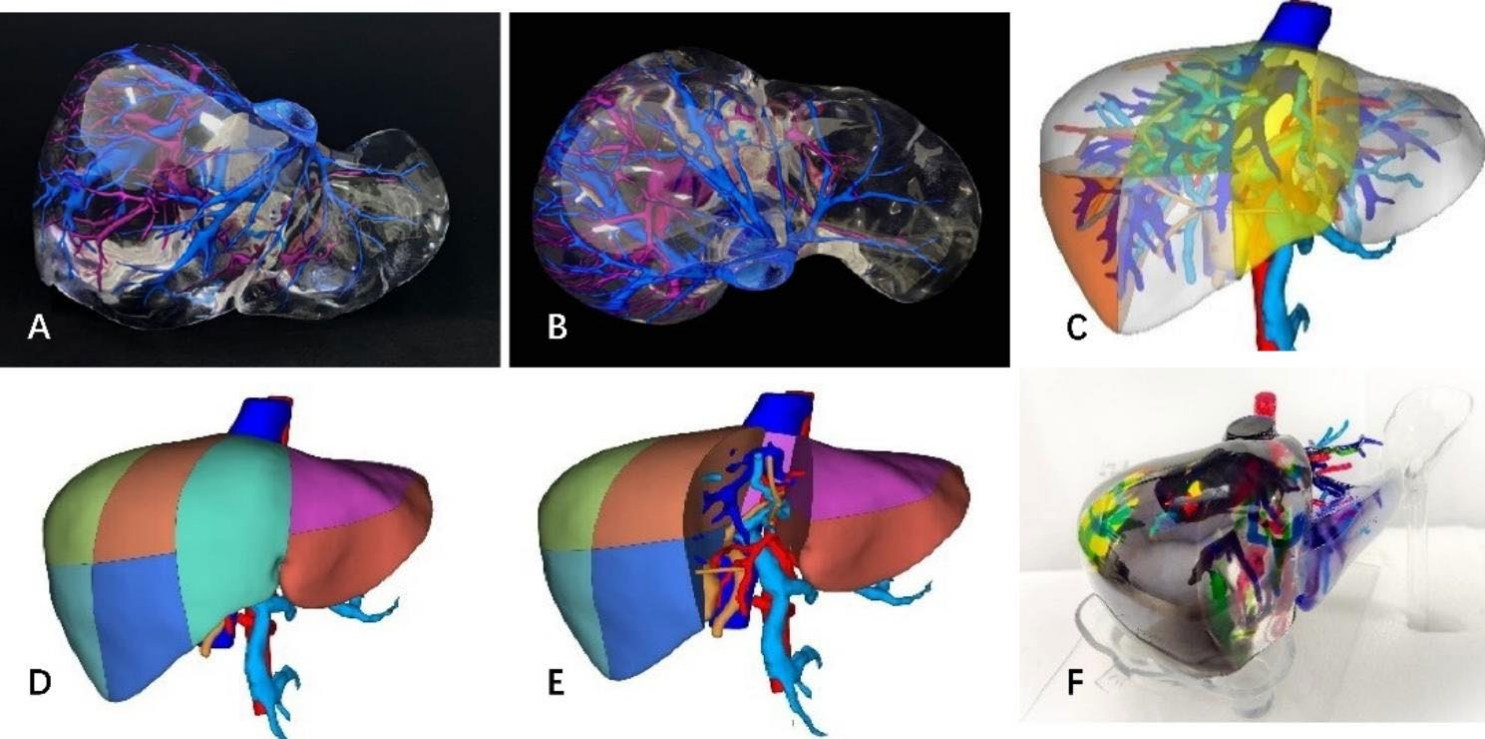
The rigid liver 3D printing model of the healthy volunteer. The parenchyma was cast using photosensitive polymer in layers with ease of visualisation of the vascular structures: the venous structure (blue) and the artery tree (pink). **A**: Anterior view. **B**: Superior view. **C**: Digital 3D reconstruction of liver lobulation and internal duct structure. **D**/**E**: Digital 3D reconstruction shows liver segment features from the front. **F**: The rigid liver 3D printing model shows liver segment features with different colours

**Fig. 2:**
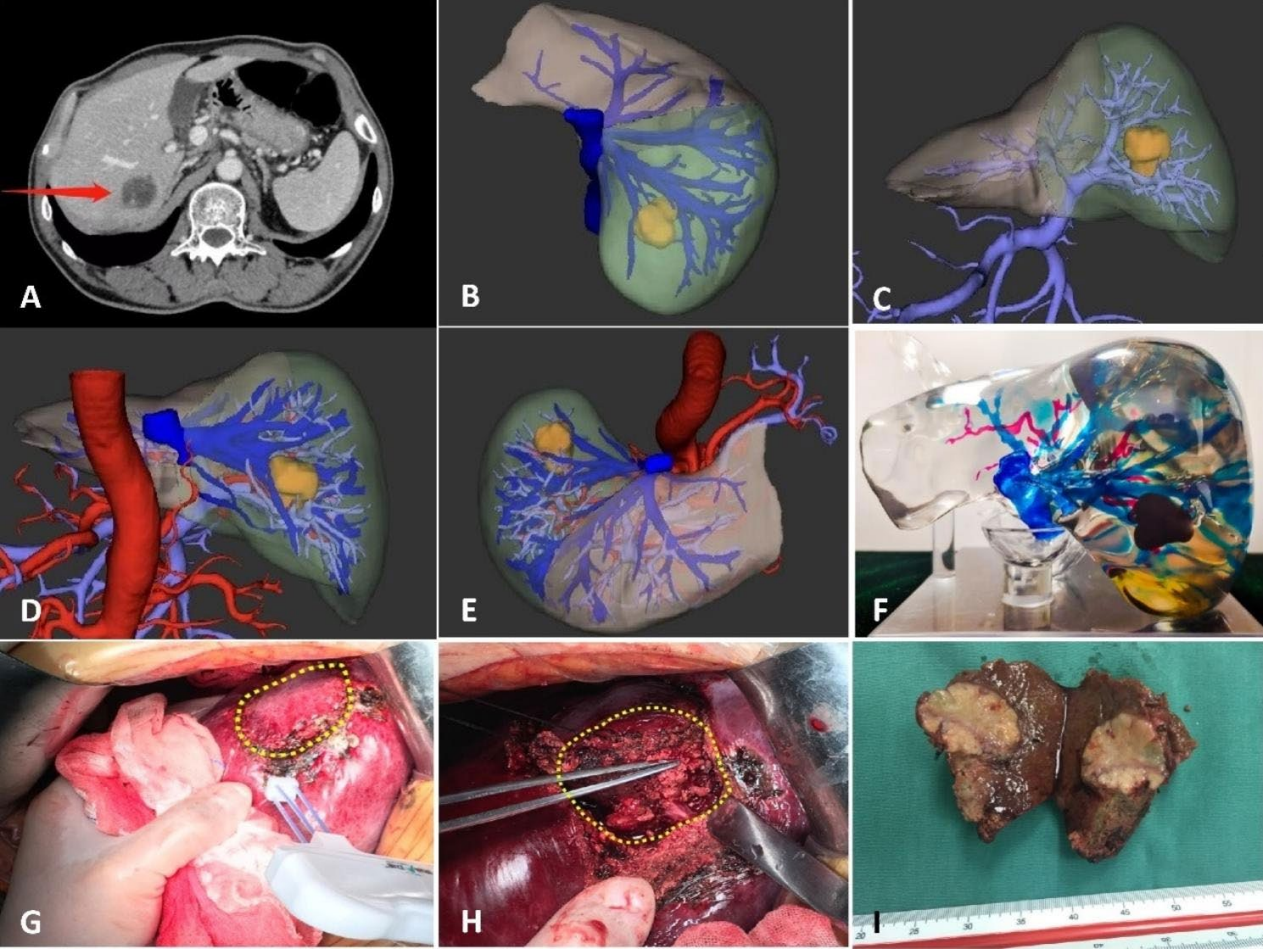
Soft liver and tumour 3D printing model of one patient. The parenchyma was cast using photosensitive polymer in layers allowing an easy cut during preoperative planning. CT images of the cross-sectional venous phase showed the tumour (**A**) and based on *CT* images (**B** Relationship between hepatic vein and tumour, **C** Relationship between portal vein branch and tumour, **D** dorsal view, **E** Superior view); The rigid liver 3D printing model (**F**) and Intraoperative image of the patient (**G**: The resection line was marked with radiofrequency ablation needle, **H**: The right posterior branch of the portal vein was preserved, **I**: Photograph of resected specimen of tumour)

The liver digital model was printed using the PolyJet3D technology of the Stratasys J850 printer with VeroCyanV/VeroYellowV/VeroMagentaV/VeroPureWhite/VeroUltraClea as the model transparent material. According to the requirements, the printing temperature was adjusted between 72 and 76 °C, and the UV light wavelength was 365 nm. Photosensitive resin material with different colours was selected for printing different anatomical structures, i.e., the gallbladder (green), portal vein (violet), artery (red), and hepatic veins (blue), as shown in Figs. 1 and 2. Noncontact resin was used as the support material for jet moulding, and it was cleaned by high-speed water flushing. The minimum thickness of the printing layer can be up to 0.016 mm, and the printing accuracy can be up to 0.1 mm. It takes approximately 14 h to print a liver model by spraying multiple photosensitive resin materials of different colours onto the same plane, and the cost of printing materials is approximately $260 USD, which can be reduced further as the number of prints increases.

### Data analysis

The number of correct responses to the questions in Questions #1 and #4 was used as the endpoint. The selected number was used as the endpoint in Questionnaires #2 and #3. Number of correct response variables of their answers to specific questions and correlations between the different gender of each group are presented with absolute frequency and were compared with the Pearson’s chi-square test or Fisher’s exact test when appropriate. The median score of the evaluation was expressed as the median [Min-Max] and analyzed by Student’s t test or Wilcoxon tests. Statistical significance was defined as a *P* value < 0.05 with a two-tailed test. Statistical analyses were performed using SPSS Statistics version 21.0 (IBM Corporation, Armonk, NY, USA).

### Ethical statement

An informed consent form was provided to the trainees of each level and the supervisors involved in the survey, and the survey was conducted after ensuring that all members understood the importance of voluntary participation and acquiring written informed consent from each participant. In addition, this study was approved by the Ethics Committee of the Tangdu Hospital of Air Force Medical University (No. K202207-06) and conducted according to the standards stipulated in the Declaration of Helsinki. Moreover, in this study, the were collected from. Written consent also was obtained for one patient and one healthy volunteer and their imaging data were anonymized.

## Results

### No improvement of trainees’ understanding of the base knowledge

**Fig. 3:**
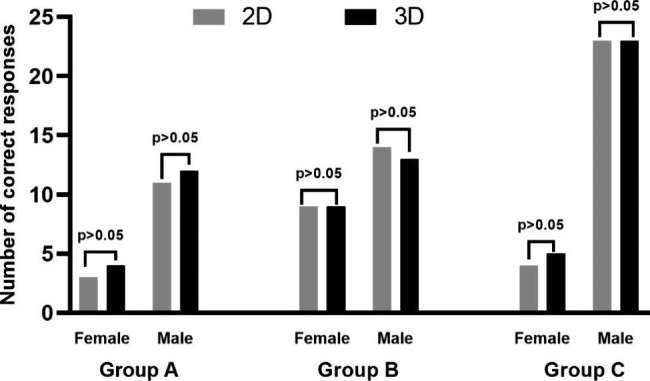
Number of correct responses on the baseline knowledge assessment across different groups with different genders

All the trainees had completed relatively standardized clinical theory courses and were given a systematic basic knowledge review together before the training. Regarding Questionnaire number 1, there was no significant difference in the number of correct responses between the different groups (Table 3). All the trainees showed good performance in answering the physiological and anatomical knowledge questions about the liver, and there was no significant difference between different training models and different genders(Fig. 3).

**Table 3:** Different levels of trainees’ understanding assessment on the base knowledge between different training models

	Number of correct responses with different genders						χ2	*P*
Group ^a^	2D			3D				
	Total	Female	Male	Total	Female	male		
A (n = 4*5)	14	3	11	16	4	12	0.533	0.465
B (n = 5*5)	23	9	14	22	9	13	0.222	0.637
C (n = 6*5)	27	4	23	28	5	23	0.218	0.640

a: According to Table S1, each trainee answers 5 questions about basic knowledge, so the total number of questions answered varies for each group: 20 for group A, 25 for group B and 30 for group C

### Significant improvement of trainees’ understanding of the key points of surgery

The trainees were asked to identify the segment(s) in which the tumour was located. The combination of 3DP models with 2D CT scans led to an improvement in tumour allocation to the liver segment in all the groups (*p* < 0.05, respectively). The trainees with fundamental experience (Group B + C) showed better performance than the interns (Group A) the combination of 3DP model training (*p* < 0.05) (Table 4). In contrast, the trainees may have recognized a tumour situated within segment VII but would fail to demonstrate exactly where the tumour is localized in a liver model.

**Fig. 4:**
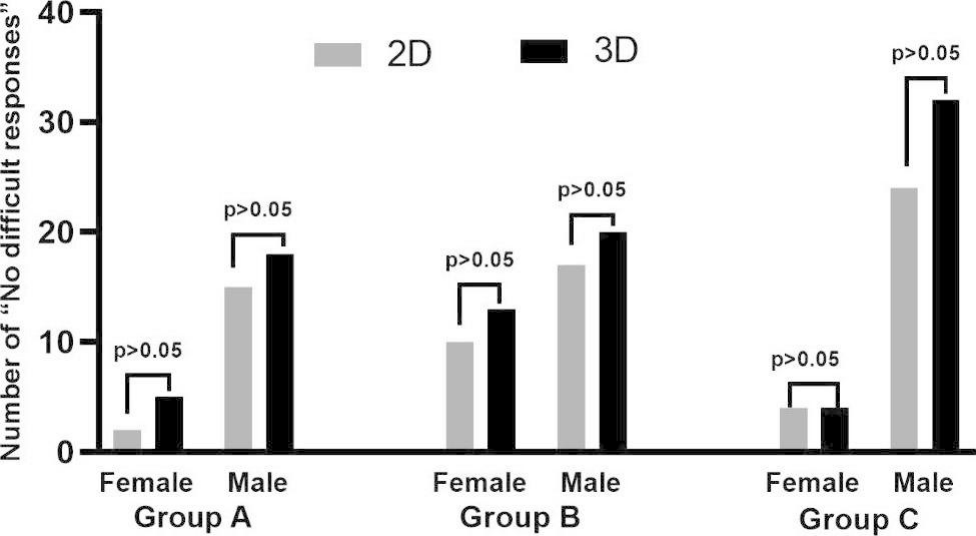
Number of “No difficult responses” on the key points of surgery of different groups with different genders

The responses of trainers of different genders in each group were compared separately. We found that in group C, the 3DP model was more helpful in improving male trainees’ understanding of the key points of surgical procedures (*p* < 0.05). However, this difference was not observed in the other two groups (Fig. 4).

The trainees were asked to draw their resection proposal line for each patient in the 3DP realistic liver model. During this test, the trainees were asked to give the minimal resection proposal, including the tumour, the safety margin, and the dependent liver tissue. Alternatively, the trainees could also proceed in a classic way by resecting the whole liver segment(s). We calculated the average percentage of the correct target area found and the number of answers that achieved more than 80% of the actual target area composed of the tumour, safety margin, and dependent liver tissue. The results are stratified according to the groups. A significant and measurable improvement could be demonstrated for the 3D presentations.

**Table 4:** Different levels of trainees’ understanding assessment of the key points of surgery between different training models

Group ^a^	Number of “No difficult responses” with different genders													χ2	P
	2D						3D								
	Total			Female		Male	Total			Female			Male		
A (n = 4*7)	17		2		15		23		5			18		4.139	0.042
B (n = 5*7)	27		10		17		33		13			20		4.200	0.040
C (n = 6*7)	28	4			24		36		4			32		4.200	0.040

a: According to Table S1, each trainee answers 7 questions about the key points of surgery, so the total number of questions answered varies for each group: 28 for group A, 35 for group B and 42 for group C

### Evaluation of the field with help to the trainees

**Fig. 5:**
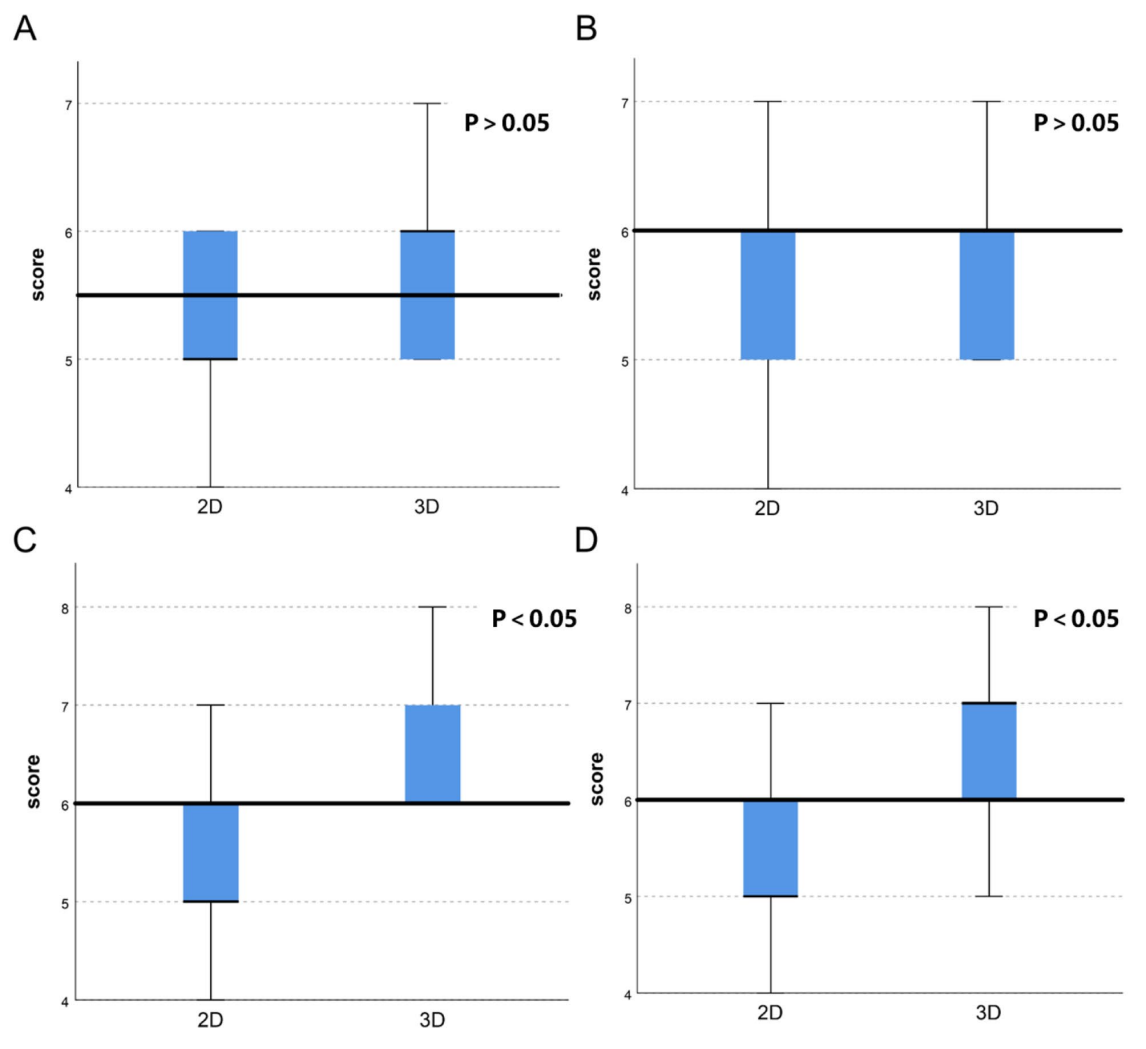
Score of correct responses per trainee, conventional 2D CT scans and 3DP model presentation. Individual analysis of trainees’ understanding improvement (number of correct responses) in four areas: A basic liver physiology and anatomy; B disease and tumour characteristics; C planned surgical procedure; D key details of the surgical procedures. A *p* value less than 0.05 was considered statistically significant

The major fields were analyzed in which 3DP models provided more help to the trainees. Although more trainees considered 3DP models helpful for their understanding of basic knowledge, including the liver itself and the tumour, no significant difference was found between 3DP models and 2D. In contrast, in the field associated with surgery and surgical details, 3DP models showed a significant advantage effect on the trainees, such as in planned surgical procedures (*p* < 0.05) and key details of the surgical procedures (*p* < 0.05) (Table 5; Fig. 5).

**Table 5:** Median score of the evaluation with help to the trainees

Items	Median [Min-Max] score of trainee’s responses		*t/Z*	*p*
	2D	3D		
Basic liver physiology and anatomy	5 [4–6]	6 [5–7]	3.333	0.068
Disease and tumour characteristics	6[4–7]	6[5–7]	1.154	0.283^a^
planned surgical procedure	5[4–7]	6[6–8]	3.968	0.046
key details of the surgical procedures	5[4–7]	7[4–7]	5.000	0.025

The score was given using a visual scale from 1 to 10 with 1 = “no help at all” and 10 = “of a great help” (“Appendix 2”). For each cognitive component, the score of the trainee’s responses reflects the level of understanding

^a^ Wilcoxon tests

### Evaluation of the monitors’ satisfaction level with the trainees’ cooperation in the operation

**Fig. 6:**
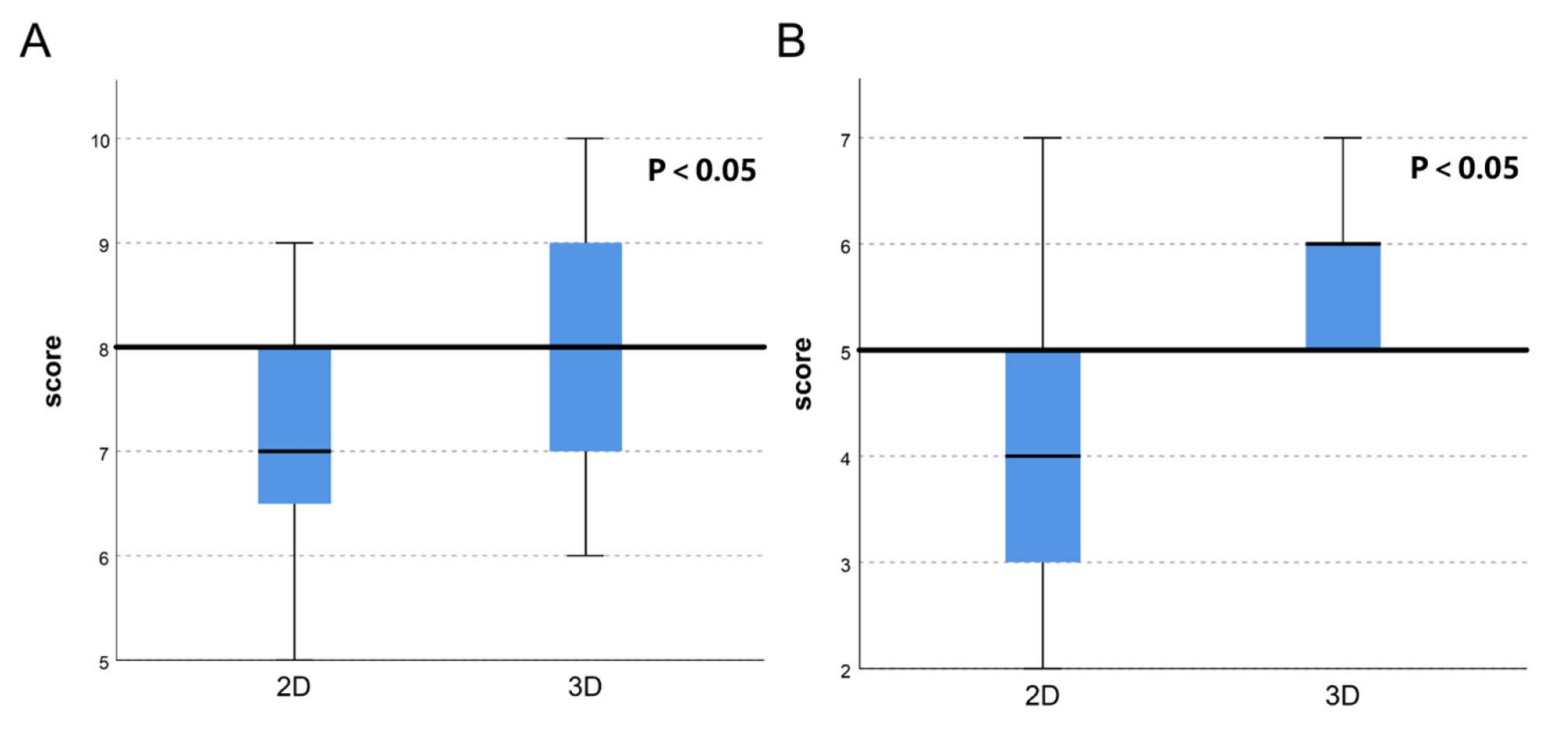
Score of correct responses per trainee, conventional 2D CT scans and 3DP model presentation. Individual analysis of monitors’ satisfaction on two areas: **A** master’s level of basic knowledge; **B** performance in assisted surgery; **A***p* value less than 0.05 was considered statistically significant

The control degree of basic knowledge and the assistance level during the operations were evaluated by the monitors. For the trainees given the hand-on 3DP model training, their supervisors gave them a better evaluation of their mastery degree of basic knowledge (*p* < 0.05). The supervisors also gave them better evaluation performance in assisted surgery (*p* < 0.05) (Table 6; Fig. 6).

**Table 6:** The median score of the monitors’ satisfaction level for the trainees

Items	Median [Min-Max] score of monitors’ satisfaction		*t*/*Z*	*p*
	2D	3D		
Master level of basic knowledge	7[5–9]	8[7–10]	3.968	0.046
Performance in assisted surgery	4[2–7]	6[3–9]	5.000	0.025^a^

The score was given using a visual scale from 1 to 10 with 1 = “No satisfaction” and 10 = “Of great satisfaction” (“Appendix 3”). For each evaluation item, the score among the group of trainees reflects the monitors’ satisfaction level

^a^ Wilcoxon tests

### Evaluation of the trainees’ satisfaction level on the training project

**Fig. 7:**
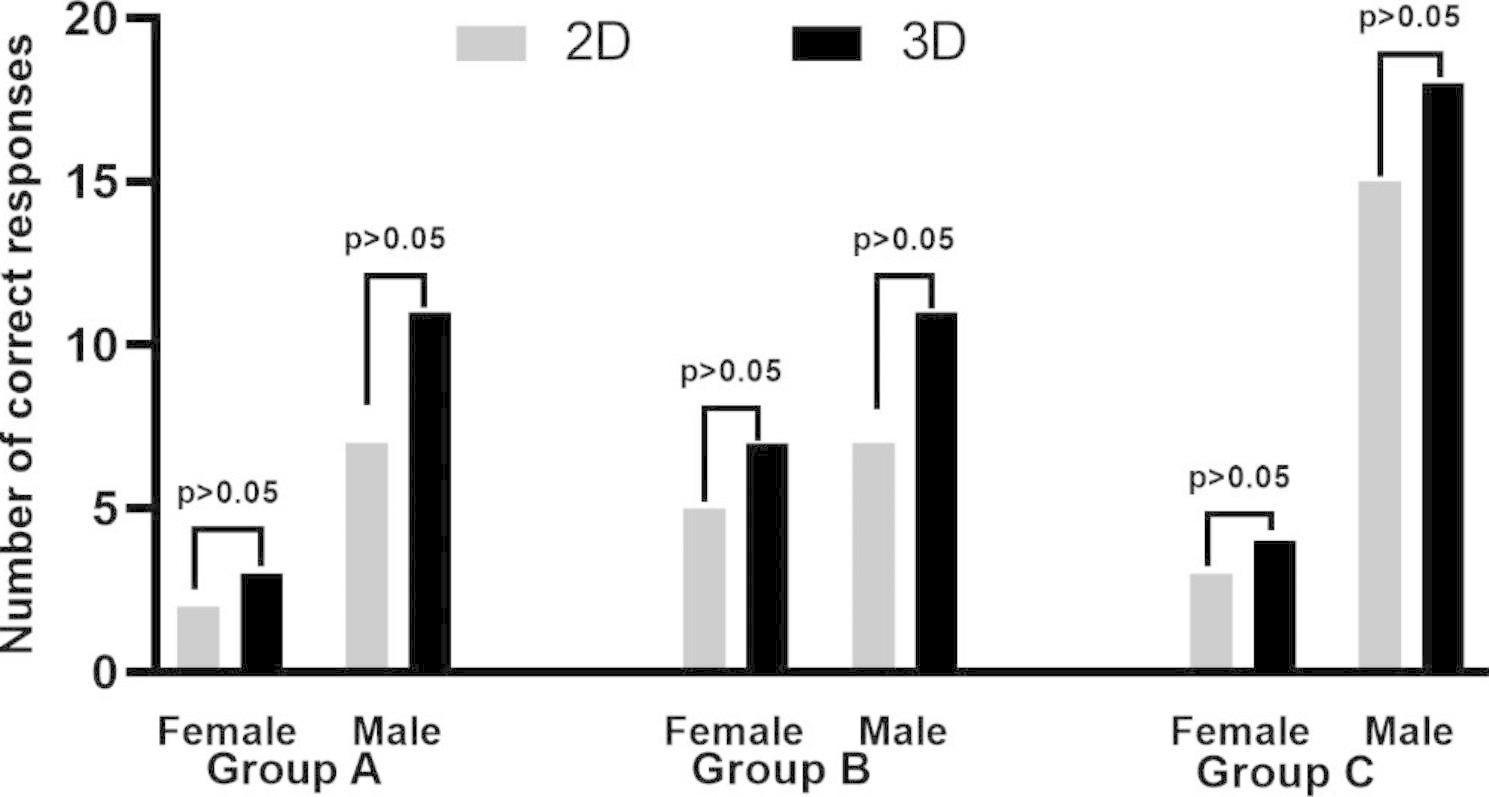
Number of correct responses of the trainees’ satisfaction level on the training project of different groups with different genders

Finally, we analyzed the evaluation of the trainees on the training project. According to Questionnaire number 4, we found that the combination of 3DP model trainees had higher satisfaction with the teaching course in all groups (*p* < 0.05). For trainees with different experience levels, their teaching satisfaction was also significantly improved with the combination of 3DP liver models with conventional 2D scans (Table 7). Most trainees agreed with the usage of the 3DP model again and agreed with its usage as a training and testing tool. Furthermore, 73% (11/15) of trainees considered that they benefitted from the project technically.The satisfaction of trainers of different genders in each group was compared separately. The results showed that gender had no effect on satisfaction evaluation in the different training models across all trainers (Fig. 7).

**Table 7:** Evaluation of the trainees’ satisfaction level with the training project

Group ^a^	Number of correct responses with different genders													χ2	*p*
	2D						3D								
	Total			Female		Male	Total			Female			Male		
A (n = 4*4)	9		2		7		14		3			11		3.865	0.049
B (n = 5*4)	12		5		7		18		7			11		4.800	0.028
C (n = 6*44)	18	3			15		22	4			18			4.547	0.033

a: According to Table S2, each trainee answers 4 questions about the training project, so the total number of questions answered varies for each group: 16 for group A, 20 for group B and 24 for group C

## Discussion

3D printing includes a wide range of technologies and applications in the fields of health care and medical education [46]. Successive improvements in 3D imaging and 3DP models have progressively led various surgical specialties to embrace these innovative technologies, especially in the field of reconstructive surgery and SBME [47, 48]. SBME has recently received a great deal of attention due to the increasing use of 3D printing technology and 3DP models in continuing medical education and clinical training [49]. Understanding complex anatomical pathology and pre- and intraoperative sets represents a critical challenge for clinical medical education [50, 51]. 3DP models can provide precise and individualized 1:1 physical models that can help trainees easily understand actual liver tumour characteristics, including tumour size, depth, and location with respect to the arteriovenous and bile duct systems, thereby contributing to preoperative surgical procedure simulation and intraoperative guidance [52].

First, in the present study, based on SBME theory, we successfully produced a full-sized physical 3DP liver model for continuing medical education evaluation using a solid transparent material, in which context the tumour and other features can be seen from the outside. Our subjective results indicated overall improvement and better performance in assisted surgery in terms of both test scores and questionnaires following the 3DP liver model training. The post-training test results of the 3D group were higher than those of the conventional 2D group. This finding is consistent with the results of previous studies [1, 49, 53]. Langridge et al. [54] used descriptive statistical methods to report the role of 3D printed models in surgical education. However, there was no difference in the mastery of basic knowledge between the two groups. Li et al. [55] did not find any significant difference (p = 0.0508) in knowledge acquisition between 3DP models and manipulatable 3D imaging. In contrast, Lim et al. [56] found that 3DP models were superior to cadavers with regard to learning external cardiac anatomy. Further evidence is thus required to determine which aspects of the 3DP models facilitate learning and whether these models could provide a superior alternative to cadavers in traditional anatomical teaching [54]. A unique feature of this study is that both self-assessment by questionnaire and rating by monitors were used to evaluate the effect of training.

Second, in addition to facilitating knowledge acquisition with regard to anatomy, 3DP liver models can also be used for surgical procedures, as they convey the anatomical relationships between organs and the surrounding tissues [57].Accordingly, our results demonstrate that the use of 3DP liver models can significantly improve all trainees’ understanding of the key points of surgery, especially for experienced male trainees. This finding further reflects the value of using 3DP liver models in continuing medical education and clinical training [58]. The evaluation of the questionnaires also showed that the participants were very satisfied overall with training on the 3DP models. Participants of all levels of experience agreed that they would benefit from such training opportunities in the future, thereby confirming the acceptance of this type of training. It was also found that providing trainees with full-sized realistic 3DP models of the liver is superior to 2D anatomy teaching methods, which is consistent with the findings of previous studies [32, 33]. The use of 3D printing technology to develop surgical simulation models and training devices has proven to offer valuable resources to clinical trainees in surgical training exercises, contribute to understanding surgical procedures, and improve surgical performance in terms of enhancing training quality and reducing learning curves [34, 59].

Finally, we investigated the gender of the trainees as an influencing factor of the training effect, and the results showed that after training with the 3DP liver models, male trainees performed better in terms of their understanding of base knowledge than female trainees, especially in the group of interns; however, there were no significant differences in the group of trainees in standardized training and the group of trainees in professional training. Vaccarezza M [60] reported that both male and female students spent less time answering questions regarding base knowledge on the spine models in the 3D group than did a conventional group. The different results found in the above research may be due to the variations in trainees experience and organs. The 3DP model was more helpful in improving male trainees’ understanding of the key points of surgical procedures and performed better in terms of cooperation with the surgical procedure. Although the number of participants included in this study was small, the results are consistent with those reported by previous studies [41, 61−62]. These findings may be due to the fact that female trainees exhibited less procedural experience and lower confidence with regard to performing procedural skills than their male counterparts despite their equal or superior performance [62–64]. In a study of fourth-year medical students, females reported overall lower levels of experience and confidence performing technical skills than males [41]. Moreover, some study also indicated that gender was an independent factor affecting spatial learning ability, a male advantage in visual-spatial working memory [39] and show faster spatial learning in a virtual environment as compared to females [40].

Compared with the recently developed multiuser virtual reality application [65], 3DP liver models offer different advantages with regard to improving resection planning for vascular reconstruction localization of resection plains, thus reducing the risks for devascularization and complications and facilitating intraoperative detection of small and deeply located tumours [66]. The study reflected the potential benefits that 3DP liver models offer in clinical medicine education compared to traditional educational tools. The results proved that 3DP models are highly suitable for training purposes in this field [67–69]. Skill-specific simulation training and other interventions may improve skill development in medical students in light of the obstacles they face when developing proficiency in the clinical setting [70].

### Limitations

However, both this study and the 3D printed training model have certain limitations. First, with regard to the design and statistical analysis of the study, the nonrandomized controlled trial and the self-reported nature of the data are the two biggest limitations. Meanwhile, this study only included thirty trainees, a sufficiently robust sample size has not been achieved, the number of participants in each group was relatively small, and the levels of anatomical education exhibited by the participants in the studies were not assessed prior to using the models, which may have introduced bias; Thus, a large-scale trial is necessary in the future. The results and findings of the study presented here should be viewed as preliminary data to support a larger and more comprehensive study featuring high-level medical training. This research could be enhanced by the inclusion of more longitudinal follow-up of participants who are exposed to 3DP models to assess their long-term skill acquisition.

Second, existing simulator 3DP models have several limitations. The most common is the material properties of the liver analogue are not physically accurate, and 3DP models have been unable to fully simulate the differences in deformation elasticity and biomechanical characteristics of human tissues. Therefore, the loss of these characteristics may limit the model’s ability to reflect both the mechanical characteristics and surface smoothness of normal tissue and may significantly affect the perceptions of and feedback provided by trainees. Furthermore, although these models replicate the procedure well, there are still few liver disease models; for example, the background of liver cirrhosis could not be effectively illustrated by the current printed models. 3D printing technology can only capture major vasculature structures [71]. Small vessels and perforators cannot be illustrated with current technology. In addition, most importantly, only a few typical liver models were printed in this study, and more complex models were not presented because of their high costs [72, 73]; since such models are an emerging technology, this situation is also a barrier to widespread clinical adoption.

## Conclusion

We have successfully created a liver 3DP model to help improve the learning effect for standardized residency training trainees and junior residents in terms of practical skills and theoretical understanding. This study supports the claim that training with 3DP models could facilitate all trainees’ presurgical understanding of liver tumours and surgery. Meanwhile, 3DP liver models of real production can serve as an effective auxiliary teaching tool for SBME teaching of Chinese standardized residency training and continuing medical education.

### Electronic supplementary material

Below is the link to the electronic supplementary material.

Supplementary Material 1

## Data Availability

All data generated or analyzed during this study are included in this published article and its supplementary information files.
