# Supplementary material for: Full-sized realistic 3D printed models of liver and tumour anatomy: a useful tool for the clinical medicine education of beginning trainees
Source: BMC Med Educ. 2023 Aug 15;23:574. doi: 10.1186/s12909-023-04535-3 (PMC10428657; doi:10.1186/s12909-023-04535-3)
Supplement: Supplementary file 1 — Supplementary Material 1 [file 12909_2023_4535_MOESM1_ESM.docx]

**Appendix 1**

Table.S1 “3D PRINTING TRAINING” study

**Questionnaire number 1. T**rainee #

| 1/the knowledge that I know about the liver | False | True | N/A |
| --- | --- | --- | --- |
| 1.1-The liver is the largest gland in the body, weighs from 1100 to 1700 grams, is a dark red color, a spongy mass of wedge-shaped lobes and occupies the upper right portion of the abdominal cavity immediately below the diaphragm. |  |  |  |
| 1.2-The liver is a large very vascular glandular organ that secretes bile and causes important changes in many of the substances contained in the blood (as by converting sugars into glycogen which it stores up until required and by forming urea). |  |  |  |
| 1.3-The liver is divided by fissures into five lobes, and that receives blood both from the hepatic artery and the portal vein and returns it to the systemic circulation by the hepatic veins. |  |  |  |
| 1.4-The liver secretes about 800 to 1,000 ml of bile per day, which contains bile salts needed for the digestion of fats in the diet. Bile is also the medium for excretion of certain metabolic waste products, drugs, and toxic substances. From the liver, a duct system carries bile to the common bile duct, which empties into the duodenum of the small intestine and which connects with the gallbladder, where it is concentrated and stored. |  |  |  |
| 1.5-The liver tumor includes benign and malignant neoplasm. HCC is the major malignant tumor. To the patients with liver tumor, hepatectomy is the fundamental treatment if it is available. |  |  |  |
| 2/the understanding that I know about the planning surgery | No difficult | Some difficult | difficult |
| 2.1-The precise site of tumor location(segment) |  |  |  |
| 2.2-The available planning resection line of the liver |  |  |  |
| 2.3-The adjacent pipe structures of the tumor |  |  |  |
| 2.4-The blood vessels and bile ducts need to be cut off |  |  |  |
| 2.5-The blood vessels and bile ducts need to be preserved |  |  |  |
| 2.6-The key details of the operative procedures |  |  |  |
| 2.7-The self-confidence for participating the operation assistance |  |  |  |

**Appendix 2**

**Questionnaire number 2. T**rainee #

Please, rate from 1 to 10(1=no help at all ->10=of a great help) to what extent the presentation of 3D liver model helper you in:

2.1-Learning about the liver itself:

**1 2 3 4 5 6 7 8 9 10**

**No help at all Of a great help**

2.2-Learning about the liver tumor:

**1 2 3 4 5 6 7 8 9 10**

**No help at all Of a great help**

2.3-Understanding the surgery you will participate:

**1 2 3 4 5 6 7 8 9 10**

**No help at all Of a great help**

2.4-Understanding the key details of the surgical procedures:

**1 2 3 4 5 6 7 8 9 10**

**No help at all Of a great help**

**Appendix 3**

**Questionnaire number 3.** **T**rainee #

Please, rate from 1 to 10(1=no satisfaction at all ->10=of a great satisfaction) to what extent of your satisfaction to the trainees:

3.1-Master level of basic knowledge of trainees:

**1 2 3 4 5 6 7 8 9 10**

**No satisfaction Of a great satisfaction**

3.2-Degree of cooperation of trainees during the operation:

**1 2 3 4 5 6 7 8 9 10**

**No satisfaction Of a great satisfaction**

**Appendix 4**

Table.S2 “3D PRINTING TRAINING” study

**Questionnaire number 4. T**rainee #

| 4/the evaluation that I know about the training project. | True | False | N/A |
| --- | --- | --- | --- |
| 4.1-I would like to use this simulation again. |  |  |  |
| 4.2-I agree with the overall value of the simulator as a training tool. |  |  |  |
| 4.3- I agree with the overall value of the simulator as a testing tool. |  |  |  |
| 4.4- I would benefit technically from this training project. |  |  |  |
